# Modulatory Effect of Nimbin on Isoproterenol Induced Mitochondrial and Lysosomal Enzymes Activities and Apoptosis Signaling in Rats

**DOI:** 10.33549/physiolres.935530

**Published:** 2025-10-01

**Authors:** Yang WANG, Jingli CHEN, Haitao JIANG, Xiaoqin LI

**Affiliations:** 1 Department of Cardiovascular Medicine, First People’s Hospital of Linping District, Hangzhou Zhejiang, China; 2 Renal Internal Medicine Dialysis Room, The Southwest Hospital of Army Medical University, Chongqing, China; 3 Department of Cardiovascular Medicine, Chifeng Municipal Hospital, Chifeng Inner Mongolia, China; 4 Department of Nephrology, Xiangyang Central Hospital, Affiliated Hospital of Hubei University of Arts and Science, Xiāngfan, Hubei, China

**Keywords:** Myocardial infarction, Tricarboxylic acid (TCA) cycle enzymes, Cathepsin-D, Cardiac markers, Triterpenoid

## Abstract

Nimbin, a bioactive triterpenoid compound isolated from the neem tree (*Azadirachta indica*), is known for its anti-inflammatory, antioxidant, antimicrobial, and hepatoprotective properties. The study aimed to explore the impact of nimbin on cardiac markers, mitochondrial and lysosomal enzyme activities, as well as apoptotic signaling in rats induced with isoproterenol. The subcutaneous injection of isoproterenol (ISO) at a dosage of 85 mg/kg body weight over the last two consecutive days led to notable increased in the activities/levels of the cardiac markers, lysosomal glycohydrolases and cathepsins. Conversely, reductions in the functioning of mitochondrial tricarboxylic acid cycle enzymes and respiratory chain enzymes in ISO-induced rats. In ISO-induced rats, there was an augmentation in the expressions of Bax, caspase-3, caspase-9, and cytochrome c, along with a diminished level of Bcl-2. Administration of nimbin resulted in decreased activities/levels cardiac markers, lysosomal glycohydrolases, cathepsins and increased functioning of mitochondrial tricarboxylic acid cycle enzymes and respiratory chain enzymes. Additionally, decreased expressions of Bax, executioner caspases and cytochrome c, along with heightened expression of Bcl-2, were noted in rats treated with nimbin. This indicates that nimbin possesses cardioprotective properties and mitigates mitochondrial and lysosomal dysfunction in rats induced with ISO.

## Introduction

Cardiovascular disease (CVD) encompasses a range of conditions affecting the heart and blood vessels, including hypertension, atherosclerosis, heart failure, stroke, and congenital heart defects [1]. By 2030, it is forecasted that CVDs will emerge as the primary reason for mortality in India, with an estimated annual death toll of 23.3 million individuals [2]. It is noteworthy to highlight that myocardial infarction (MI) remains the primary contributor to cardiovascular diseases [3]. MI arises due to an elevated demand for myocardial metabolism and a reduced delivery of oxygen and nutrients to the myocardium through the coronary circulation, resulting in cellular damage [4,5].

In the present research, ISO, a catecholamine, was administered, serving as a standard model for investigating the positive impacts of various drugs on cardiac function. Previous studies suggest that subjecting the heart to increased catecholamine levels can lead to generation of necrotic tissue damage within the heart muscle of intervention animals [6]. Catecholamines experience quick self-oxidation, and there is a suggestion that the oxidative derivatives of catecholamines play a role in the alterations observed in the myocardium. The ISO-induced myocardial necrosis resulted in modifications to membrane permeability, leading to the impairment of myocardial membrane function and integrity [7,8].

Mitochondria serve as the primary provider of cellular energy within our body. Various factors and substances have the potential to negatively impact the functioning of mitochondria. The leakage of lysosomal enzymes into the intracellular fluid is a significant factor in acute myocardial infarction [9]. Lysosomes are a group if cytoplasmic organelles present in numerous animal tissues that contain hydrolytic enzymes capable or degrading the cellular constituents. The release of these enzymes into the cytoplasm stimulates the formation of inflammatory mediators such as oxygen radicals and prostaglandins [10]. Lysosomal leakage can play an initiative role in apoptosis induced by oxidative stress. In myocardial ischemia, the increased activities of glycohydrolases usually decrease the lysosomal stability, resulting in necrosis of myocardium [11]. Decker *et al.* have reported that ISO-treated myocardial necrosis involves membrane permeability alterations that bring about loss of function and integrity of myocardial membranes [12].

Numerous medicinal plants are widely recognized for their ability to alleviate inflammatory conditions, including diabetes, infections, and cancer. Neem (*Azadirachta indica*) stands out as a medicinal plant with significant potential for addressing a variety of human health issues, including cardiovascular diseases. Neem has demonstrated its efficacy in treating various health disorders such as skin conditions, diabetes, dental and oral issues, as well as gastric ulcers, among others [13–17]. Nimbin sourced from neem, such as triterpenoids, and flavonols extracted from various sections of the botanical specimen, are attracting attention for their potential in addressing various diseases [18,19]. Nimbin, an active component of the neem tree, has been identified to possess diverse pharmacological activities. Mittal *et al.* conducted a study indicating that nimbin effectively interacted with the active sites of protein kinase B (Akt) and phosphatidylinositol 3-kinase (PI3K), exhibiting binding energies were found to be −8.2 and −6.8 kcal/mol, respectively [20]. According to Sharma *et al.* neem seed oil was identified to contain nimbin, which triggers apoptosis and halts the cell cycle in breast cancer [13]. Sharma *et al.* demonstrated that the combined encapsulation of doxorubicin and nimbin displayed heightened anticancer efficacy against THP-1 cells when subjected to near-infrared (NIR) light exposure [21]. Sudhakaran *et al.* reported that analogs of nimbin inhibit adipogenesis by suppressing reactive oxygen species (ROS) and superoxide anion while enhancing glutathione levels in zebrafish larvae exposed to a high-fat diet [22]. As per Sudhakaran *et al.* nimbin exhibited cytotoxic effects on MG-63 osteosarcoma cells, restraining cell proliferation and migration. It induced mitochondrial dysfunction, activated the caspase cascade, and stimulated apoptosis, ultimately resulting in programmed cell death [23].

The focus of this study was to examine the defensive properties of nimbin against isoproterenol-induced cardiac ischemia by assessing alterations in cellular respiratory organelles and intracellular degradation organelles in rat models. The study employed rat models to investigate nimbin’s influence on myocardial mitochondrial and lysosomal enzymes when exposed to ISO-induced myocardial infarction. Additionally, we validated the impact of nimbin on apoptosis in a rat model induced with ISO.

## Materials and Methods

### Animal model

To assess the cardioprotective effect of nimbin, experiments were conducted using Swiss Albino male rats weighing 160–180 g, aged 5–7 weeks. The rats were obtained from Xiangyang Central Hospital and maintained in suitable conditions, receiving a standard diet with unrestricted access to drinking water. The research adhered to approved guidelines, and all parameters were scrutinized in compliance with the Institutional Animal Ethics Committee’s recommendations.

### Experimental induction of MI

To initiate myocardial infarction (MI), a dosage of 85 mg/kg body weight (b. wt.) of ISO was suspended in isotonic saline solution (0.9 % NaCl) and introduced *via* subcutaneous injection (s.c.) into the rat’s right thigh. This process was repeated for two consecutive days, with a 24-hour interval between administrations.

### Study design

The experimental setup involved four groups, each comprising six rats.

Group I – Control
Group II – Oral administration of nimbin (10 mg/kg b.wt.) for a period of 21 days
Group III – ISO control (85 mg/kg b.wt. at s.c.) for the last two consecutive days
Group IV – Oral administration of nimbin (10 mg/kg b.wt.) for a period of 21 days + ISO control (85 mg/kg b.wt. at s.c.) for the last two consecutive days.

The experiment concluded with the euthanasia of rats after an overnight fasting period. Rats were sedated through an intramuscular injection of ketamine. The blood drawn *via* cardiac puncture was collected and subsequently left to clot naturally at room temperature. The serum was prepared before being centrifuged at 400× g, 10 min. The cardiac tissue was taken immediately, washed with saline and used for various analysis.

### Serum biomarkers for MI evaluation

Serum samples were utilized to assess the enzymatic functions of CK-MB, cTnT, and cTnI. Commercial reagents obtained from Xiamen Boson Biotech Co., Ltd, Xiamen were employed for the measurement of CK-MB, cTnT, and cTnI activities, following the manufacturer’s instructions.

### Assessment of cardiac mitochondrial enzyme activity through tissue extraction

Mitochondrial-rich fractions were obtained from the heart tissue of various experimental animals using a method of differential centrifugation, as described in previously documented research. In summary, a homogenate with a concentration of 20 % (w/v) was formulated using a mixture of 0.25 M sucrose, 0.05 M Tris-HCl buffer, and 5.0 mM EDTA. The first centrifugation at 600× g for 10 min efficiently separated cellular debris, tissue remnants, and cell nuclei, resulting in the formation of a nuclei pellet. Following this, the supernatant was subjected to centrifugation at 10000× g for 5 min at 4 °C, resulting in the formation of the mitochondrial pellet. The remaining pellet, predominantly composed of healthy mitochondria, was carefully retained, while the upper part, potentially containing loose material, was discarded. The removal of lipid-rich foamy material was crucial to prevent uncoupling of mitochondria. Upon reconstitution of the mitochondrial pellet in a defined volume of potassium chloride (KCl), it was employed to determine multiple parameters, including the functionality of cardiac mitochondrial enzymes such as isocitrate dehydrogenase (ICDH) [24], succinate dehydrogenase [25], malate dehydrogenase (MDH) [26], α-ketoglutarate dehydrogenase (α-KGDH) [27], cytochrome c oxidase [28] and nicotinamide adenine dinucleotide dehydrogenase [29] were analyzed.

### Isolation and assessment of lysosomal enzyme activity from cardiac tissue

The lysosomal fraction from the heart tissue was extracted following the procedure outlined by DePierre and Karnovsky [30]. Initially, fresh tissue was homogenized in ice-cold 0.25 M sucrose, followed by centrifugation at 3000× g and 15000× g. The resulting lysosomal pellet was resuspended in 1.15 % KCl for subsequent enzyme analysis, including β-glucuronidase [31], α and β-glucosidase [32], α and β-galactosidase [33], β-N-Acetyl glucosaminidase [34], Cathepsin-B [35], and Cathepsin-D [36] were analyzed.

### Quantitative Real-Time RT-PCR in cardiac sample

Heart tissue-derived total RNA, extracted using TRIzol reagent, was quantified by measuring absorbance at 260 and 280 nm on a Biophotometer (Eppendorf). Subsequently, the RNA underwent reverse transcription into cDNA following established protocols. Real-time PCR was conducted in a StepOne Plus thermocycler (Applied Biosystems) using Power SYBR Green master mix, adhering to the manufacturer’s instructions. Transcript expression levels of the target genes were standardized against glyceraldehyde 3-phosphate dehydrogenase (GAPDH). The experiments were conducted independently three times, and the assessment of relative mRNA expression employed the 2^−ΔΔCt^ technique.

### Western blot analysis

The heart tissues were washed with cold 1×PBS and then lysed for 30 min at 4 °C in RIPA buffer with protease inhibitor. Following lysis, the cell lysates were centrifuged for 10 min at 4 °C at 15000× g. The protein samples (30 μg/lane) were loaded and electrophoresed on SDS-PAGE. By electroblotting, proteins were moved from the gel onto a nitrocellulose membrane. A 5 % bovine serum albumin-blocking solution was used to block the membrane protein for 2 h. Following blocking, TBST was used twice to wash the membrane. The membranes were incubated for 4 h at 4 °C with the 1^st^ Ab (Bax, Caspase-3, -9, Cytochrome-c, Bcl-2 and GAPDH) and washed twice with TBST and following probed with specific HRP-conjugated the 2^nd^ Ab at 37 °C for 2 h. In the end, TBST was used to wash the membranes twice times. The protein bands were developed by chemiluminescent substrate.

### Statistical analysis of the data

Statistical analyses were carried out using one-way analysis of variance (ANOVA), and comparisons between groups were performed using Duncan’s Multiple Range Test (DMRT) *via* the SPSS/21 Software Package. The findings were reported as mean ± standard deviation for each group, with six rats in each group. Statistical significance was determined at a P-value below 0.05.

### Ethical approval statement

The animal ethical approved by the Institutional Animal Ethics Committee’s in the Xiangyang Central Hospital, Affiliated Hospital of Hubei University of Arts and Science, Xiāngfan, Hubei, China.

## Results

### Impact of nimbin on cardiac markers

ISO-induced groups showed that the elevation in serum concentration of CK, CK-MB, cTnT, cTnI and LDH. Nevertheless, pretreatment with nimbin restored all these cardiac indicators to baseline levels in ISO-induced rats (Table 1).

### Evaluation of TCA enzyme response in ISO-induced rat hearts under the influence of nimbin

Mitochondrial dysfunction plays a significant role in the onset of various cardiovascular diseases (CVDs). To assess the effect of nimbin on tricarboxylic acid (TCA) cycle enzymes in rat heart tissue, we analyzed the activity of key enzymes crucial for mitochondrial function. Table 2 presents the mitochondrial enzyme activities in both the control and treatment groups. The enzymes involved in the oxidative metabolism of pyruvate in mitochondria – such as isocitrate dehydrogenase (ICDH), alpha-ketoglutarate dehydrogenase (α-KGDH), succinate dehydrogenase (SDH), and malate dehydrogenase (MDH) – showed reduced activity in rats with myocardial infarction (MI) compared to the control group. However, rats pretreated with nimbin at a dose of 10 mg per kilogram of body weight exhibited a significant increase in enzyme activity compared to the ISO-induced group.

### Effect of nimbin on lysosomal enzymes activities in control and ISO-induced rat hearts

Table 3 depict the performance of lysosomal enzymes in the hearts of control and ISO-induced rats. A marked increase was observed in the activities of β-glucosidase, α- and β-galactosidase, β-glucuronidase, cathepsin B, and cathepsin D in the cardiac muscle of rats with ISO-induced cardiac injury. However, in rats pretreated with nimbin, there was a significant reduction in the activity of these acid hydrolases in myocardial tissue compared to those treated with ISO alone.

### Effect of nimbin on apoptotic gene expression in control and ISO-induced rats

**Fig. 1:**
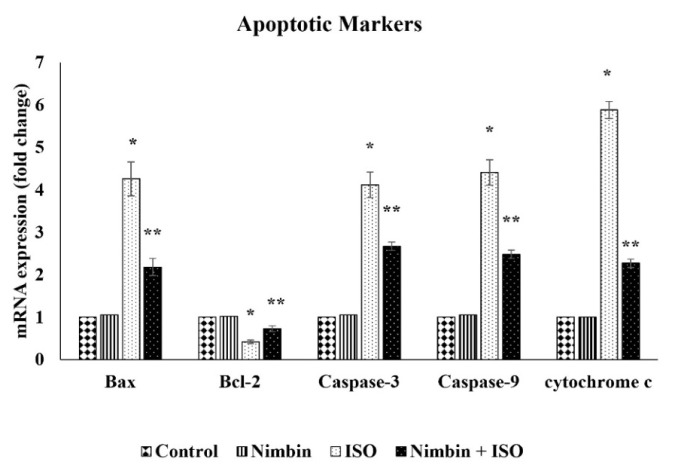
Effect of nimbin on apoptotic gene expressions in heart tissue of control and ISO-induced experimental rats. The histogram depicts the quantitation of three independent experiments (means ± SD). The Bax, Bcl-2, caspase-3, −9 and cytochrome c genes expression levels were normalized with the expression level of the GAPDH mRNA in each sample. Values are expressed as means ± SD for three rats in each group. * Significantly different from the control group (*p*<0.05). ** Significantly different from the ISO group (*p*<0.05).

To investigate the impact of nimbin on pro- and anti-apoptotic molecules in Figure 1. In heart tissue, there was a decline in Bcl-2 expression and an elevation in Bax, cytochrome c, caspase-9, and -3 expression in rats stimulated with ISO compared to reference rats. However, the application of nimbin to ISO-induced rats resulted in an upregulation of Bcl-2 expression and attenuation of Bax cytochrome c, caspase-9, and −3 expression when compared to ISO-induced rats.

### Effect of nimbin on ISO-induced apoptotic protein expression in heart tissue

**Fig. 2:**
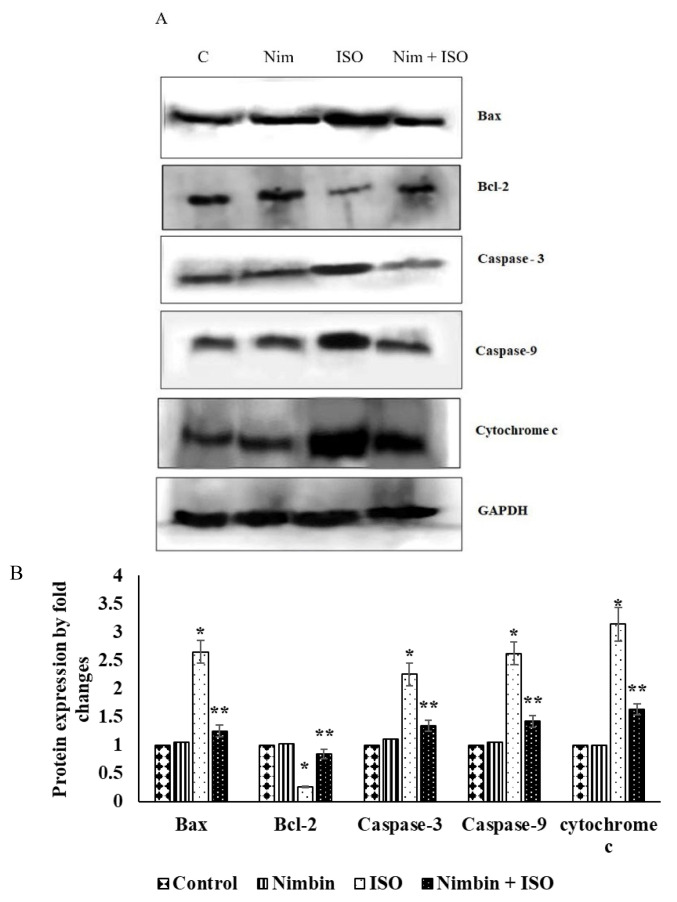
Effect of nimbin on apoptotic protein expressions in heart tissue of control and ISO-induced experimental rats. (**A**) Effect of nimbin on ISO-induced Bax, Bcl-2, caspase-3, −9 and cytochrome c protein expression in heart tissue by Western blot analysis. (**B**) Densitometry analysis of apoptotic markers normalized by GAPDH. Values are expressed as means ± SD for three rats in each group. * Significantly different from the control group (*p*<0.05). ** Significantly different from the ISO group (*p*<0.05).

Cardiac apoptotic markers, including Bax, Bcl-2, caspase-3, −9 and cytochrome c were analyzed using Western blotting. In ISO-induced rats, the expression of these markers was significantly increased compared to control rats. However, treatment with nimbin markedly inhibited the expression of Bax, Bcl-2, caspase-3, −9 and cytochrome c in ISO-treated rats, as shown in Figure 2.

## Discussion

Myocardial infarction poses a significant clinical challenge linked to cardiac damage, and the available therapeutic approaches are limited and often accompanied by notable side effects. Consequently, the effectiveness of various medications in providing cardioprotection is assessed using an experimental model induced by ISO to mimic myocardial infarction [37]. Many investigations have indicated that the physiological, structural, and metabolic shift observed in the hearts of intervention animals after the application of ISO closely resemble those seen in humans. The induced myocardial infarction by ISO stands as a standardized model for examining the positive impacts of various drugs on cardiac functions [38,39].

Studies have indicated that incorporating plant-based dietary agents can lower the susceptibility to cardiovascular diseases [40]. Nimbin, a natural compound derived from *Azadirachta indica*, has been traditionally utilized for the treatment of various ailments [41]. The focus of this research was to assess the cardioprotective effectiveness of nimbin against ISO-induced myocardial infarction by evaluating cardiac markers, as well as mitochondrial and lysosomal enzymes, along with examining apoptosis.

This investigation demonstrated a significant increase in myocardial markers, including CK-MB, cTn T, and cTn I, in the blood serum of ISO-infused rodents, in alignment with results documented by previous reported [42,43]. Under physiological circumstances, CK-MB is usually contained within cellular compartments, with its release into the bloodstream occurring following myocardial injury, as a result of the degradation of myofibrils and sarcotubular system [44]. Troponins T and I are precise polypeptide found in myocardial tissue, forming part of the troponin complex crucial for cardiomyocyte damage. These cardiomyocyte troponins serve as remarkable specific and discerning indicators of myocardial trauma, making them the ideal markers for diagnosing cardiac infarction [45,46]. The administration of nimbin led to a significant reduction in CK-MB activity and lowered the levels of cTnT and cTnI. This effect may be attributed to nimbin’s ability to preserve membrane stability; this consequently limits the discharge of these cardiac markers into the bloodstream.

Mitochondria, vital subcellular structures responsible for energy generation, are susceptible to oxidative stress. Given their abundance in cardiomyocytes, these organelles play a crucial role in myocardial injury. Exploring the interactions between drugs and mitochondria as secondary targets can enhance our understanding of the underlying mechanisms. Mitochondrial dysfunction significantly impacts the pathogenesis of various cardiovascular diseases (CVDs). Within the myocardium, mitochondria occupy approximately 45 % of the volume of the heart muscle [47]. In ischemic conditions, the generation of lipid peroxides and hydrogen peroxide initiates cascading reactions that may harm mitochondrial membranes. Matsuzaki *et al.* observed that reactive oxygen species (ROS) generated during ischemia can harm various macromolecules, including enzymes [48]. In our examination of ISO-induced rats, we noticed a reduction in the tasks of mitochondrial citric acid cycle enzymes (ICDH, SDH, MDH, and α-KGDH) within heart mitochondria. These enzymes, situated on the outer mitochondrial membrane, may be adversely impacted by the excessive generation of reactive oxygen species infused by ISO. Administration of nimbin to ISO-induced rats beforehand led to a notable increase in the activities of mitochondrial citric acid cycle enzymes. This enhancement could be attributed to nimbin’s potential to hinder the production of reactive oxygen species and its capacity to scavenge them, as evidenced by our results.

The lysosomal vesicle membrane plays an indispensable role in regulating cellular protease secretion in pathophysiological conditions and inflammation-triggered situations. Rats treated with ISO exhibited elevated actions of acid hydrolases (β-glucuronidase, α and β-galactosidase, cathepsin-B and D) in both serum and cardiac muscle. The heightened lipid peroxidation seen in rats treated with ISO could potentially result in the liberation of blood and cardiac tissue proteases from intracellular vesicles as a consequence of lysosomal membrane impairment. It has been recorded that there were increased actions of serum and cardiac acid hydrolases in rodents treated with ISO [49]. Research has proven that the release of intracellular acid hydrolases from lysosomes and the muscle cell endoplasmic reticulum contributes to the malfunction and degradation of mitochondria, cell membrane, and additional cellular structures [2]. Premedication with nimbin normalized the functions of hydrolytic enzymes by inhibiting lipid peroxidation, thereby preventing ISO-induced damage to lysosomes in rats.

Disruption of the hydrolytic enzymes fidelity may result in an unwanted rise of enzyme levels both inside and outside the cell, potentially leading to cellular and tissue abnormalities, including apoptosis. Preserving the stability of the hydrolytic enzymes is essential for regulating standard levels of intracellular hydrolases and proteases in tissues and bodily fluids. The excretion of β-glucuronidase serves as an indicator of the structural integrity of the lysosomal barrier. The decrease in β-glucuronidase and cathepsin-D actions within the lysosomal component suggests a decline in membrane stability [50,51]. The heightened enzyme activities observed in the cytosolic portion stem from the discharge of these enzymes from the lysosomal compartment into the cytoplasm in rodents exposed to ISO treatment. Pre-exposure with nimbin effectively hindered the dissemination of these enzymes from the intracellular compartment in ISO-treated rats. The redistribution of enzyme functions from the cytosolic compartment to lysosomal and cytosolic to overall enzymatic activities indicate a decrease in lysosomal stability in rats treated with ISO. The administration of nimbin prevented the excretion of acid hydrolases and reduced the overall function of intracellular hydrolytic enzymes thereby augmenting lysosomal stability. This outcome could be attributed to nimbin’s membrane-stabilizing properties.

Existing literature proposes that oxidative stress-induced apoptosis of cardiomyocytes is a key factor in causing damage to cardiac tissue and advancing the progression of myocardial infarction [52]. Proteins such as Bcl-2 play a crucial role as regulatory components, safeguarding cells from cellular suicide, while Bax and caspase-3, identified as cell death-inducing genes, actively encourage cell demise. Certainly, redox imbalance triggers apoptosis networks by increasing the expression of Bax cellular factor and caspase protease, while concurrently reducing the levels of the antiapoptotic Bcl-2 [53,54]. This analysis reinforces the hypothesis by revealing a substantial rise in the regulation of apoptotic markers and cell death-associated genes and a diminished levels of anti-apoptotic factor – Bcl-2, following ISO treatment juxtaposed to the reference group. This occurrence has the potential to elevate apoptosis and lead to functional irregularities in the heart muscle. The halting caspase and Bax activation stands out as a significant strategy for mitigating myocardial apoptosis [55]. The administration of nimbin effectively restrained oxidative stress through its antioxidant properties. Additionally, nimbin treatment led to an elevation of Bcl-2 genes in the myocardial tissue, while simultaneously downregulating the expression of proapoptotic genes and apoptosis-related genes, Bax, caspase-3, −9 and cytochrome c in rats with ISO-stimulated myocardial infarction. This protective action helped safeguard myocardial cells from apoptosis.

## Significance

The significance of this study demonstrates the cardioprotective effect of nimbin on myocardial infarction induced by ISO in rats. Nimbin’s ability to protect heart tissue from damage caused by myocardial infarction, a condition where blood flow to the heart is severely reduced or blocked. Nimbin helps maintain the structural integrity of mitochondria and other intracellular organelles. Potential effect of nimbin to prevent apoptosis suggests it could help in preserving heart cells that would otherwise die in response to ischemic damage. This anti-apoptotic effect is crucial in mitigating the extent of myocardial injury and improving recovery outcomes. Overall, the study underscores nimbin’s promising role in heart health, specifically in counteracting damage from myocardial infarction and supporting cellular repair and survival.

## Conclusions

The study outcomes suggest that nimbin exhibits cardioprotective properties in rats with ISO-induced myocardial infarction by maintaining the structural integrity of both mitochondria and Intracellular digestive organelle membrane. Furthermore, nimbin restores enzyme activities to levels approximating normalcy in rats exposed to ISO-induced conditions. Additionally, nimbin demonstrates potential in preventing apoptosis, indicating its efficacy in averting apoptotic alterations. Hence, supplementation with nimbin appears advantageous in alleviating myocardial infarction induced by ISO.

## Figures and Tables

**Table 1:** Effect of nimbin on cardiac markers in the serum of control and ISO-induced in experimental rats.

Groups	Control	Nimbin	ISO	Nimbin + ISO
*CK (IU/l)*	150.83 ± 17.92^a^	149.47 ± 12.68^a^	251.76 ± 20.41^b^	181.27 ± 22.61^c^
*CK-MB (IU/l)*	92.43 ± 8.37^a^	90.28 ± 9.98^a^	167.32 ± 17.19^b^	121.27± 10.19^c^
*cTnT (ng/ml)*	0.78 ± 0.07^a^	0.78 ± 0.07^a^	01.72 ± 0.19^b^	01.10 ± 0.18^c^
*cTnI (ng/ml)*	0.58 ± 0.06^a^	0.56 ± 0.04^a^	01.12 ± 0.14^b^	0.71 ± 0.05^c^
*LDH (IU/l)*	198.23 ± 18.05^a^	197.78 ± 17.09^a^	284.27 ± 28.22^b^	219.17 ± 19.34^c^

Data are expressed as means ± SD for six rats in each group. The data not sharing a common superscript (a, b, c) are different significantly at *p*≤0.05 (DMRT).

a No significant difference between control and Nimbin group.

b Significantly different from the control group (*p*<0.05).

c Significantly different from the ISO-induced group (*p*<0.05).

**Table 2:** Effect of nimbin on the activities of mitochondrial TCA cycle enzymes and respiratory chain enzymes in the heart of control and ISO-induced rats.

Parameters	Control	Nimbin	ISO	ISO + Nimbin
*Mitochondrial TCA cycle enzymes*				

*ICDH (U* *^#^* */mg protein)*	785.15 ± 52.22^a^	784.38 ± 62.18^a^	358.27 ± 57.49^b^	687.28 ± 61.08^a^
α-KGDH (U*/mg protein)	88.32 ± 7.84^a^	87.81 ± 7.93^a^	46.41 ± 4.27^b^	71.63 ± 6.84^c^
*SDH (U* *^♣^* */mg protein)*	206.82 ± 18.24^a^	204.38 ± 10.19^a^	164.76 ± 12.38^b^	180.94 ± 16.83^c^
*MDH (U* *^♦^* */mg protein)*	344.81 ± 31.98^a^	348.34 ± 4.56^a^	226.47 ± 19.39^b^	305.47 ± 29.19^c^

*Respiratory chain enzymes*				

*Cytochrome c oxidase (U* *^♠^* */mg protein)*	0.529 ± 0.108^a^	0.404 ± 0.102^a^	0.218 ± 0.118^b^	0.447 ± 0.059^a^
NADH dehydrogenase (U**/mg of protein)	128.24 ± 10.77^a^	129.83 ± 10.21^a^	75.29 ± 8.33^b^	108.29 ± 8.73^a^

Values are expressed as means ± S.D. for six rats in each group. Values not sharing a common superscript (a, b, c) differ significantly at *p*<0.05 (DMRT). U^#^=nmol of α-ketoglutarate liberated/hour, U*=nmol of ferrocyanide formed/hour, U^♣^=nmol of succinate oxidized/minute, U^♦^=nmol of NADH oxidized/minute, U^♠^=change in O.D/minute, U**=nmol of NADH oxidized/minute.

a No significant difference between control and Nimbin group.

b Significantly different from the control group (*p*<0.05).

c Significantly different from the ISO-induced group (*p*<0.05).

**Table 3:** Effect of nimbin on the activities of lysosomal enzymes in the heart tissue of control and ISO-induced rats.

Parameters	Control	Nimbin	ISO	ISO + Nimbin
* Heart*				
*α-Glucosidase (U* *^♣^* */100 mg protein)*	14.28 ± 1.53^a^	14.58 ± 1.39^a^	32.28 ± 4.66^b^	18.83 ± 1.72^c^
*β-Glucosidase (U* *^♣^* */100 mg protein)*	8.17 ± 1.12^a^	8.33 ± 0.88^a^	21.29 ± 1.76^b^	11.59 ± 1.17^c^
*α-Galactosidase (U* *^♣^* */100 mg protein)*	7.39 ± 0.48^a^	7.41 ± 0.64^a^	13.82 ± 1.15^b^	9.27 ± 0.89^c^
*β-Galactosidase (U* *^♣^* */100 mg protein)*	15.41 ± 0.81^a^	15.64 ± 1.43^a^	29.27 ± 1.83^b^	18.69 ± 1.93^c^
*β-Glucuronidase (U* *^♣^* */100 mg protein)*	9.29 ± 0.84^a^	9.41 ± 0.89^a^	18.37 ± 1.76^b^	11.47 ± 1.04^c^
*b-N-Acetyl glucosaminidase (U* *^♣^* */100 mg protein)*	36.91 ± 2.72^a^	38.37 ± 3.07^a^	58.51 ± 5.05^b^	40.47 ± 3.23^a^
Cathepsin B (U*/100 mg protein)	22.14 ± 2.09^a^	23.14 ± 2.73^a^	44.73 ± 4.23^b^	29.08 ± 2.06^c^
*Cathepsin D (U* *^♦^* */100 mg protein)*	17.29 ± 1.76^a^	17.43 ± 1.52^a^	27.62 ± 1.94^b^	19.56 ± 1.79^c^

Values are expressed as means ± S.D. for six rats in each group. Values not sharing a common superscript differ significantly at *p*<0.05 (DMRT). U^♣^=μmol of p-nitrophenol liberated/hour, U*=μmol of p-nitroaniline liberated/hour, U^♦^=μmol of tyrosine liberated/hour.

a No significant difference between control and Nimbin group.

b Significantly different from the control group (*p*<0.05).

c Significantly different from the ISO-induced group (*p*<0.05).

## Abbreviations

α-KGDH: alpha-ketoglutarate dehydrogenase
Bax: Bcl-2-associated X protein
Bcl-2: B-cell leukemia/lymphoma-2
B.wt.: Body weight
CK: creatine kinase
cTnI: cardiac troponin I
cTnT: cardiac troponin T
CVD: cardiovascular disease
GAPDH: glyceraldehyde 3-phosphate dehydrogenase
ICDH: isocitrate dehydrogenase
ISO: isoproterenol hydrochloride
MDH: malate dehydrogenase
MI: myocardial infarction
ROS: reactive oxygen species
SDH: succinate dehydrogenase
TCA: tricarboxylic acid
